# Identification of Inhibitors of Integrin Cytoplasmic Domain Interactions With Syk

**DOI:** 10.3389/fimmu.2020.575085

**Published:** 2021-01-08

**Authors:** Deenadayalan Bakthavatsalam, John W. Craft, Anna Kazansky, Nghi Nguyen, Goeun Bae, Amy R. Caivano, C. William Gundlach, Asra Aslam, Safa Ali, Shashikant Gupta, Sophie Y. Lin, Hema D. Parthiban, Peter Vanderslice, Clifford C. Stephan, Darren G. Woodside

**Affiliations:** ^1^ Molecular Cardiology Research Laboratories, Texas Heart Institute, Houston, TX, United States; ^2^ Department of Biology and Chemistry, University of Houston, Houston, TX, United States; ^3^ Center for Translational Cancer Research, Institute of Biosciences and Technology, Texas A&M Health Science Center, Houston, TX, United States

**Keywords:** inflammation, cell adhesion, integrin, signaling, tyrosine kinase, immune response receptor, high-throughput screening, β-lactam antibiotics

## Abstract

Leukocyte inflammatory responses require integrin cell-adhesion molecule signaling through spleen tyrosine kinase (Syk), a non-receptor kinase that binds directly to integrin β-chain cytoplasmic domains. Here, we developed a high-throughput screen to identify small molecule inhibitors of the Syk-integrin cytoplasmic domain interactions. Screening small molecule compound libraries identified the β-lactam antibiotics cefsulodin and ceftazidime, which inhibited integrin β-subunit cytoplasmic domain binding to the tandem SH2 domains of Syk (IC_50_ range, 1.02–4.9 µM). Modeling suggested antagonist binding to Syk outside the pITAM binding site. Ceftazidime inhibited integrin signaling *via* Syk, including inhibition of adhesion-dependent upregulation of interleukin-1β and monocyte chemoattractant protein-1, but did not inhibit ITAM-dependent phosphorylation of Syk mediated by FcγRI signaling. Our results demonstrate a novel means to target Syk independent of its kinase and pITAM binding sites such that integrin signaling *via* this kinase is abrogated but ITAM-dependent signaling remains intact. As integrin signaling through Syk is essential for leukocyte activation, this may represent a novel approach to target inflammation.

## Introduction

Integrin cell adhesion molecules are expressed on all cell types and are essential for proper organism development and tissue homeostasis (1, 2). In addition, integrins play a significant role in the pathophysiology of numerous diseases including those of the immune system (3–8). Because they regulate key steps in leukocyte recruitment into tissue, including leukocyte tethering, firm adhesion, diapedesis, and migration (9), integrins have been directly implicated in inflammation and autoimmunity. Furthermore, integrins not only provide adhesive contacts, but also serve as signaling molecules that are essential for leukocyte activation within sites of inflammation (10, 11).

Originally identified as a key signaling component of the B-cell antigen receptor and Fc-receptors (12), the non-receptor spleen tyrosine kinase (Syk) is now recognized as an essential integrin signaling component in inflammation (13, 14). One of the earliest events in integrin signaling is the activation of Syk (15–17). Integrin signaling *via* Syk is involved in neutrophil spreading, respiratory burst and degranulation (11), costimulation of the expression of interleukin (IL)-1β in monocytes (18), and extracellular signal-regulated protein kinase activation in macrophages (10, 19).

Integrin activation of Syk is a result of the direct interaction between integrin cytoplasmic domains and the N-terminal SH2 domains of Syk (20, 21) that results in Syk clustering and either transactivation (22) or activation by associated src family kinases (22). Immune response receptor activation of Syk requires interaction between Syk tandem SH2 domains and immunoreceptor tyrosine-based activation motif (ITAM)-containing adaptor proteins such as DAP12 or FcRγ (13, 14, 23). Current models suggest that the direct association between integrin cytoplasmic domains and Syk allows for Syk recruitment into integrin signaling complexes that contain ITAM-bearing adaptor proteins, which results in maximal activation of Syk and downstream effectors (13, 14, 22, 23).

The integrin: Syk signaling axis is a promising area for therapeutic intervention. Identifying antagonists of integrin cytoplasmic domain interactions with Syk would provide new molecular tools to elucidate the nature of integrin and ITAM-containing adaptor molecule co-signaling through Syk in leukocyte activation. Here, we describe the development of high-throughput screening (HTS) systems that were used to identify inhibitors of integrin cytoplasmic domain interactions with Syk. These inhibitors, when incorporated into cells, impede integrin signaling through Syk but do not prevent FcγRI signaling, demonstrating that specific integrin proximal signaling pathways can be targeted while leaving other signaling pathways intact.

## Materials and Methods

### Cell Lines

#### Sources of Cell Lines

THP-1 cells were sourced from ATCC. The cell line was derived from a male source.

#### Culture and Maintenance of Cell Lines

Cell culture was performed using standard techniques per ATCC recommendations. THP-1 cells were cultured using RPMI 1640 supplemented with 10% fetal bovine serum (FBS), 1% penicillin/streptomycin (Gibco), and 0.05 µM 2-mercaptoethanol at 37°C in 5% CO_2_.

### Method Details

#### β3 Peptides

**Figure 1:**
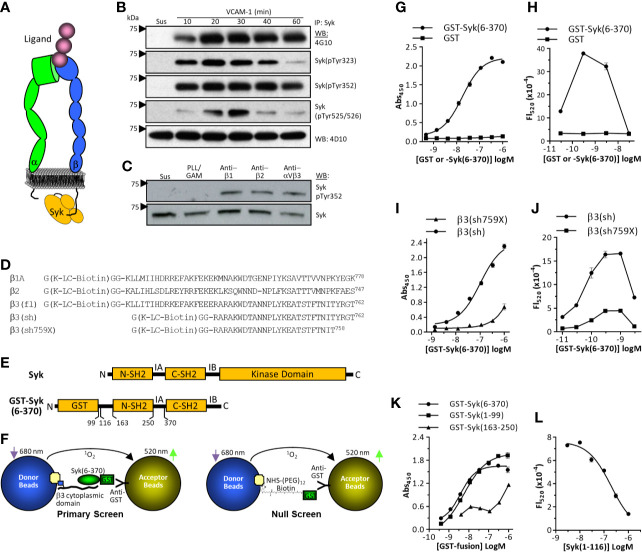
Integrin signaling *via* Syk and cell-free screen development. **(A)** Schematic representation of a ligand-bound integrin with the short intracellular β–chain cytoplasmic domain directly interacting with the tandem SH2 domains of Syk. **(B)** Integrin α4β1-dependent activation of Syk. THP-1 cells were either maintained in suspension (Sus) or plated on vascular cell adhesion molecule-1 (VCAM)-1 (immobilized at 3 ug/ml) for various time points. Cell lysates were immunoprecipitated with monoclonal antibody 4D10, then probed by western blot using indicated antibodies. One of three representative experiments is shown. **(C)** THP-1 cells were either maintained in suspension, plated on plastic immobilized poly-L-Lysine/GAM (as a non-specific adhesion control), or GAM-captured anti-β1 monoclonal antibody (mAb) TS2-16, anti-β2 mAb 76C3, or anti-αVβ3 mAb LM609. One of four representative experiments is shown. **(D)** Synthetic peptides based on the cytoplasmic domain of the integrin β1A, β2, and β3 subunits. Cytoplasmic domains were synthesized with an N-terminal region that included a biotin modified lysine residue (K-LC-biotin). Numbering is based on Uniprot canonical human sequences absent signal peptides (https://www.uniprot.org). **(E)** Schematic representation of Syk. SH2, src homology 2 domain; IA, Interdomain A; IB, Interdomain B. **(F)** Schematic representation of primary (left) and false-positive (right) AlphaScreen results. **(G, I, K)** ELISA-based β3: GST-Syk binding assays performed as described in the *Methods*. Data represent mean ± SEM from triplicate determinations; one of three representative experiments is shown. **(H, J, L)** AlphaScreen assays as described in the *Methods*. Data represent mean ± range from duplicate determinations. One of four representative experiments is shown.

Integrin β3 cytoplasmic domain peptides were synthesized with N-terminal modifications that included a dual glycine spacer, a penultimate lysine long chain biotin (LC Biotin), and an N-terminal glycine (**Figure 1D**) (24). The long (fl) and short (sh) β3 peptide sequences comprised 46 amino acids (residues 716–762) and 28 amino acids (residues 734–762), respectively, of the β3 cytoplasmic domain. These were synthesized, purified using high performance liquid chromatography (>90%), and verified by mass spectrometry (New England Peptide, MA, USA).

#### GST-Syk Constructs

Generation of glutathione-S-transferase (GST)-Syk constructs [GST-Syk(1-163); GST-Syk(1-99); GST-Syk(6-370); GST-Syk(1-116); GST-Syk(165-268)] were as follows: pGEX-4T-Syk(1-163) construct was generated from EMCV/Myc-Syk (21) as a template with forward and reverse primers listed in the Key Resources Table. The Syk(1-163) fragment that corresponds to the N-terminus SH2 (N-SH2) and IA domain of Syk was amplified by polymerase chain reaction (PCR). The PCR fragment was then digested with *EcoR*I/*Xho*I and ligated into the *EcoR*I/*Xho*I digested pGEX-4T1 (GE Healthcare) plasmid. Using this pGEX-4T-Syk(1-163) plasmid as a template DNA and the forward and reverse primers listed in the Key Resources Table, we generated a pGEX-4T-Syk(1-99) construct by inserting a stop codon (TAG) using a QuikChange II XL Site-Directed Mutagenesis Kit (200521, Stratagene) following the manufacturer’s protocol. A pGEX-4T-Syk(1-116) plasmid was also generated by inserting a stop codon in the pGEX-4T-Syk (1-163) construct, and GST-free-Syk(1-116) protein was prepared as described under Protein Purification. For the pGEX-Syk(165-268) construct, using pGEX-2T-Syk(6-370) plasmid (25) as a template DNA and the forward and reverse primers listed in the Key Resources Table, we amplified the *syk(165-258)* fragment corresponding to the C-terminus SH2 domain (C-SH2) of Syk by PCR. This fragment was cloned into a TA cloning vector, pCR2.1 (45-0046, Invitrogen), which was then digested using *Sal*I and *Not*I to cut out the *syk(165-258)* fragment and ligated into the *Sal*I and *Not*I sites in pGEX-4T1 (GE Healthcare) to construct pGEX-Syk(165-258). The resulting vectors were transformed into BL21(DE3) competent cells (69450-3, Novagen) to express and purify GST fusion protein.

#### Protein Purification

Syk expression construct-transduced BL21(DE3) cells were grown overnight at 37°C in Luria broth medium (Invitrogen) in the presence of 100 ug/ml ampicillin. The overnight culture was then diluted with LB medium to an OD600 of 0.05 and further grown at 37°C. Once the culture reached an OD600 of 0.4–0.5, protein expression was induced by adding isopropylthio-β-galactoside to a final concentration of 0.1 mM. After 3 h of induction at 37°C or overnight at 25°C, the cells were collected by centrifugation at 12,000 × *g* for 20 min and stored at −20°C. Frozen pellets were suspended (30 ml per L of culture) in phosphate-buffered saline (PBS) lysis buffer (1.8 mM KH_2_PO4, 10.1 mM Na2HPO4, 137 mM NaCl, 2.7 mM KCl, pH 7.4) with complete, EDTA-free protease inhibitors (Roche, cat#11873580001), 2 mM phenylmethylsulfonyl fluoride (PMSF), 2 mM dithiothreitol (DTT), and 1% Triton-X 100. The collected cells were disrupted through sonication (Qsonica, 50% amplitude, 15-s pulse with 30 s intervals for 3 min, repeated 3–5×). The lysate was clarified at 16,000 × *g* for 30 min, and recombinant proteins were purified from the supernatant by using Glutathione-Sepharose beads (cat# 17075601, GE Healthcare) (0.5 ml of packed beads per 1 L of culture) and were incubated overnight while rocking at 4°C. The beads were washed in 100 mM Tris pH 8.0, 100 mM NaCl, 1 mM PMSF, and 1 mM DTT, and protein was eluted using wash buffer containing 20 mM GSH. To generate GST free Syk(1-116), the GST tag was cleaved from GST-Syk(1-116) by incubating overnight at 4°C with thrombin agarose beads, as per the manufacturer’s protocol with the exception of a 200-ul reaction volume (Thrombin CleanCleave Kit, Sigma, cat#SLBF0950). The beads were then separated from the supernatant by spinning in a spin filter column (P2003-1, Zymo). The 200 ul of supernatant containing free GST and Syk(1-116) was then incubated further with 20 ul Glutathione-Sepharose beads for 1 h at 4°C (GE healthcare) to remove cleaved GST. This step was repeated until free GST was completely removed. Syk(1-116) purity was analyzed by SDS-PAGE (**Supplemental Figure S2**).

#### Biotinylated GST

We expressed and purified GST following the Protein Purification protocol above (pGEX-4T-1 in DH5α as per manufacturer’s instructions, GE Healthcare Lifesciences), and then we modified it with biotin linked through a (PEG)_3_ spacer (Solulink Sulfo ChromaLink Biotin, Solulink, cat#B-1007). Covalent protein modification is expected to occur between lysine side chains and the water soluble sulfo-NHS ester moiety. The ChromaLink reagent contains an internal bis aryl hydrazone (λ_max_ 354 nm) that allows for simple quantitation by UV-vis analysis (Thermo Scientific Nanodrop spectrometer). The molar substitution ratio was calculated from the ratio of the bis aryl chromophore concentration to protein concentration, both using Beer’s Law, and was determined to be approximately one, or, on average, one biotin per GST. Integrity of the biotinylated protein was verified by SDS-PAGE and AlphaScreen assays (**Supplemental Figures S2** and **S3**).

#### Primary and Null Amplified Luminescent Proximity Homogeneous Assay (AlphaScreen)

The following HTS assay was developed to identify antagonists of the interaction between the integrin β3 cytoplasmic domain and the non-receptor tyrosine kinase Syk. The assay uses the AlphaScreen assay format (26–30) as a measure of the binding between the integrin β3 cytoplasmic domain residues 716–762 and the tandem SH2 domains of Syk (residues 6-370). β3 peptides were synthesized with a penultimate N-terminal biotinylated lysine residue (**Figure 1D**), and Syk residues were expressed and purified as a GST-fusion protein [e.g. GST-Syk(6-370)] (**Figure 1E**). Background signals in this assay were determined by omitting the integrin β3 peptide. Specific assay conditions are described in **Table 1**. Briefly, anti-GST conjugated AlphaScreen acceptor beads (Perkin Elmer cat# 6760603) were coated with GST-Syk(6-370) and plated in Optiplate-384-well plates (Perkin Elmer). Then, either dimethyl sulfoxide control or compound was added, followed by biotinylated β3 peptide, and avidin-coated AlphaScreen donor beads. After a 4-h incubation period at room temperature (while protected from light), assays plates were read on an Infinite M1000 (Tecan) multiplate reader (Ex. 680/30; Em. 570/100).

**Table 1:** AlphaScreen Assay Parameters.

Sequence	Parameter	Value	Description
1	Plate assay buffer	10 ul	See notes for assay buffer components
2	Plate library compounds	100 nl	25 uM final concentration
2.1	Combine GST-Syk(6-370) with acceptor beads (1 h, before dispensing into assay plate)		GST-Syk(6-370) (0.3 nM); acceptor beads (10 ug/ml)
3	Plate GST-Syk(6-370) coated acceptor beads	10 ul	
4	β3 peptide	10 ul	100 nM
5	Donor beads	10 ul	10 ug/ml
6	Incubation	4 h	
7	Assay readout		
Notes			
1	Assay buffer—25 mM HEPES (pH7.4), 100 mM NaCl, 0.1% TritonX-100, 0.01% BSA; Plates used—384-well white Optiplates (PerkinElmer, cat#6007290).		
2	384-pin transfer from stock donor plates. Pins used were FP3NS100H from V&P Scientific, Inc San Diego, CA, USA.Description of pins: Floating Tube, 100nl-Slot Hydrophobic Pin—0.787 mm diameter, 38.1 mm long, 17 mm exposed pin length		
3–5	All reagents were dispensed using a Thermo Multidrop combi, sequence 3-5.		
6	TopSeal-A (cat# 6050195 from Perkin Elmer), centrifuge at 1,000 rpms for 1 min before the 4-h incubation at room temperature in the dark		
7	Plate reader with integrated, dedicated AlphaScreen module and laser light source.		

#### ELISA-Based Orthogonal Assays

For ELISA-based assays, 96-well plates (Costar cat# 9018) were coated overnight at 4°C with neutravidin (Thermo Scientific, cat#31050) in 10 mM Tris pH 9 and 100 mM NaCl. After overnight incubation, non-specific binding sites were blocked with bovine serum albumin (BSA) (2% w/v) for at least 1 h at room temperature. Plates were washed in 25 mM HEPES pH 7.5, 100 mM NaCl, 0.1% BSA, and 0.01% TritonX-100 (assay buffer), and indicated biotinylated β3 peptide was incubated for at least 2 h at room temperature (0.5–3 ug/ml in di-H_2_O). After washing in the assay buffer, GST-fusion proteins were added (50 ul, indicated concentrations) and incubated at room temperature for 1 h in assay buffer. For compound inhibition studies, compounds were added to GST-fusion proteins (at indicated concentrations) before they were added to plates. Plates were washed 3× with assay buffer (200 ul) to remove unbound GST-fusion protein. Horseradish peroxidase (HRP)-conjugated anti-GST antibody [Polyclonal anti-GST(Z-5)-HRP, Santa Cruz Biotechnology, cat #sc-459 0.2 ug/ml] was incubated in washed plates in 50 ul assay buffer for 45 min at room temperature. After washing, the signal was generated with 1-step Ultra TMB-ELISA (Thermo Scientific, cat#34028), stopped with 2 N H_2_SO_4_, and read on a plate reader (SPARK 10M*/Tecan*) at 450 nm.

#### Thermal Denaturation Assays

In this assay, Syk 6-370 (GST tag removed by thrombin cleavage) was used. Thrombin (GE Healthcare cat#270846, reconstituted in PBS to 1 unit/ul) was applied to the GST-Syk 6-370 protein bound to the Glutathione Sepharose beads. The GST-tag was cleaved with 20 unit/1 ml of protein-GST beads in PBS overnight at room temperature. After chromatography and additional cleaning on GST-beads, the purified protein was verified by SDS-PAGE. For thermal denaturation experiments, GST-free Syk(6-370) was used at a final concentration of 2 µM and SYPRO orange (Thermo Scientific, cat# S6650) at a 0.08% (v/v) dilution (from a 5000x stock). Compounds were used at indicated concentrations. Numerous reaction buffers were initially screened, and a final buffer containing 25 mM HEPES (pH 7.5) with 100 mM NaCl was used. Assays were performed in 20 µl volumes, in triplicate, in 384-well plates (Applied Biosystems MicroAmp 384-well clear optical reaction plate, catalogue #4483285). Plates were sealed with Applied Biosystems Optical Adhesive Cover (cat#4360954) and centrifuged for 2 min at 800 × g at room temperature. Thermal denaturation was measured on a Quant Studio 6 Flex Real-time PCR system (Applied Biosystems). Reaction conditions were as follows: initial 2:00 min hold at 25°C; ramp up in 1°C increments to a final temperature of 95°C; 2:00 min hold at 95°C; run method used was “Step and Hold” 1:00 min intervals.

#### Peptide Affinity Pull-Down Assays

THP-1 cells were washed in PIPES buffer (10 mM PIPES, 50 mM NaCl pH 6.8) and resuspended at 10 × 10^6^ cells/ml in PIPES lysis buffer [PIPES buffer containing 1% Triton X-100, 1 mM EDTA, 1 mM Na_3_VO_4_, 50 mM NaF, 150 mM sucrose, protease inhibitors (cOmplete, Mini, EDTA-free Protease Inhibitor Cocktail, Roche, cat#11836170001)] and 0.1 mM N-ethylmaleimide. The cells were incubated overnight in lysis buffer at 4°C with end-over-end rotation. Lysates were then passed through a 21-gauge needle and centrifuged at 13,000 × *g* for 30 min at 4°C. Lysate protein concentrations were determined by using Quick Start Bradford Dye reagent (BioRad cat#5000202). Cell lysates (100 µg/ml) were treated with or without drug (at indicated concentrations) for 2 h at room temperature before the peptide affinity assays. In parallel, 300 µl high-capacity Neutravidin agarose resin (Thermo Scientific, cat#29204) was washed once with 5 ml of PIPES lysis buffer and incubated (2 h at room temperature) in 1 ml of PIPES lysis buffer containing 250 µg of integrin β3 cytoplasmic domain peptide and 20% FBS. β3 peptide-bound beads were then washed once with PIPES lysis buffer and resuspended in 600 µl PIPES lysis buffer. Cell lysate treated with or without drug was then incubated with 100 µl β3 bound beads for 1 h at room temperature followed by washing (3×) with PIPES lysis buffer. Endogenous Syk bound to β3 peptide beads was eluted with 40 µl of reduced SDS sample buffer. Eluted samples were run on SDS-PAGE (4–20%, BioRad, cat# 4561093) and immunoblotted for Syk using anti-Syk antibody at 1:1,000 (4D10; SantaCruz) dilution followed by GAM-HRP at 1:5,000 and detected with SuperSignal West Pico (Thermo Scientific, cat# 34580). Western blots were scanned, and band intensity was measured using ImageJ (31) to compare and determine the effect of the drug on Syk/β3 binding. Beads alone were used as a negative control.

#### Glycerol Facilitated Compound Loading

Compound loading of cells was facilitated by glycerol shock (32, 33). Methods were first optimized with the cell-impermeable fluorophore Sulfo-Cy5 (see **Supplemental Figure S5**). For analysis of compound inhibition of integrin signaling, 3 × 10^6^ THP-1 cells were incubated in 200 ul serum-free RPMI containing 15% glycerol and ceftazidime (at indicated concentrations) for 1 h at 20°C. RPMI (3 ml, room temperature) containing ceftazidime (at indicated concentrations) was added to the cells to dilute the glycerol, and the cells were further incubated for 10 min (room temperature). Cells were then plated onto CS-1-BSA-coated plates for 15 min at 37°C, lysed in RIPA buffer (1% Triton X-100, 50 mM Tris-HCl pH 7.2, 150 mM NaCl, 0.25% NaDOC, 0.05% SDS, 2 mM PMSF, 1 mM NaF, 1 mM Na_3_VO_4_, and protease inhibitor cocktail) (AEBSF, Aprotinin, E-64, Bestatin, Leupeptin and Pepstatin; VWR Cat#97063-010), and the lysates were subjected to Western blot as described below.

#### Syk Phosphorylation

Syk phosphorylation assays were performed by immobilizing vascular cell adhesion molecule-1 (VCAM)-1 (3 ug/ml), CS-1-BSA (10 ug/ml), or specific monoclonal antibodies (anti-FcγRI mAb 276426, anti-α4β1 mAb 19H8, anti-β2 mAb 76C3, anti-αvβ3 mAb LM609 all at 5 ug/ml) overnight at 4°C in Tris-HCl pH 7.5, 150 mM NaCl, in 6-well plates (non-tissue culture treated, Becton Dickenson, cat# BD351146). Plates were then blocked with BSA (2% w/v) for 1 h at room temperature. In **Figure 1C**, GAM was immobilized in all wells (2 h at 37°C in 200 mM Na_2_HCO_3_ pH 9.2, blocked for 1 h in 2% BSA), followed by the addition of Poly-L-Lysine (PLL) or indicated monoclonal antibodies (5 ug/ml) in Tris-buffered saline (TBS) overnight at 4°C. After washing wells in serum-free RPMI, untreated or drug-loaded THP-1 cells (5 × 10^6^ cells per well) were plated in standard serum-free RPMI (1.5 ml total volume), allowed to adhere for 10 min at 37°C 5% CO_2,_ and then immediately lysed in RIPA buffer (1% Triton X-100, 50 mM Tris-HCl pH 7.2, 150 mM NaCl, 0.25% NaDOC, 0.05% SDS, 2 mM PMSF, 1 mM NaF, 1 mM Na_3_VO_4_, and protease inhibitor cocktail) (AEBSF, Aprotinin, E-64, Bestatin, Leupeptin and Pepstatin; VWR Cat#97063-010). Cells kept in suspension served as non-adherent controls and were lysed under the same conditions. For Syk immunoprecipitations, 5 × 10^6^ THP-1 cell equivalents were incubated overnight at 4°C with monoclonal antibody 4D10 at 4 ug/mg protein and were rotated. After overnight incubation, protein G-sepharose (20 ul packed beads washed in RIPA) (GE Healthcare cat# 17061801) was added to lysates, which were incubated for 2 h at 4°C, while rotating. Sepharose was washed 3× in RIPA by centrifugation, pelleted, and then resuspended in SDS-PAGE sample buffer (reducing). For whole cell lysates not subjected to immunoprecipitation, approximately 1 × 10^6^ cell equivalents were loaded for SDS-PAGE under reducing conditions (4–20%, Thermo Scientific, cat#25204). After electrophoresis, proteins were transferred to nitrocellulose for 1 h 100 V room temperature, blocked in 5% non-fat milk in TBS-T, and probed with the indicated primary antibody at 1:1,000 overnight at 4°C, followed by species-appropriate HRP secondary antibody (Southern Biotech) at 1:5,000 (30 min at room temperature), and detected using Pierce West Pico (Thermo Scientific cat# 34580). For blot stripping, nitrocellulose was subjected to 0.4 N NaOH for 20 min at room temperature, blocked as described above, and re-probed with the indicated antibodies (primary antibody at 1:1,000 for 1 h at room temperature, followed by HRP-conjugated secondary antibody at 1:5,000 for 30 min at room temperature).

#### Quantitative PCR

THP-1 cells (2 × 10^6^ per treatment group) that were glycerol loaded with ceftazidime (100 µM) or vehicle control (as described above) were plated on CS-1 peptide (10 µg/ml; 6-well plates) in serum-free RPMI-1640. Control cells were also kept in suspension. Plated and suspension cells were incubated for 4 h at 37°C. After incubation, the medium was removed, and RNA was isolated from cells with a Qiagen RNA extraction kit. After cDNA synthesis, quantitative real-time PCR was used to determine the relative gene expression levels of IL-1β and monocyte chemoattractant protein (MCP)-1 in THP-1 cells adhered to CS-1 in the presence of vehicle or ceftazidime relative to that of cells in suspension. The cycle threshold (Ct) results generated were analyzed according to the comparative Ct method (2^-ΔΔCt^), where ΔCt is the difference between the CT value of target *versus* GAPDH control, and ΔΔCt is the difference between the ΔCt values of a given treated adherent cell sample and that of cells in suspension. Statistical differences between the two treatment groups for each target were determined by a two-tailed, unpaired Student *t* test using GraphPad Prism 7.04 statistical software.

### Modeling

All docking was done in triplicate for both large and focused search boxes and was initialized with random seeds. Autodock Tools was used to prepare ceftazidime for docking into the input molecular model of SYK, prepared using standard protocols (34, 35) from crystallographic structure 4FL2 (36). Docking was completed with Autodock Vina 1.1.2 (37, 38) using an initial unbiased search box (**Supplementary Figure S7**) with center (−11.6, 3.6, 34.0) Å and size (90,80,80) Å and then refined by using a focused box encompassing the putative binding sites, identified from the first round. Resultant PDBQT files were split into each PDB using split2pdb and Babel. TCL scripts were used to automate running VMD post processing and generating MATLAB input for KMeans analysis in either four or six bins. Clusters for each bin were then analyzed to generate heat maps with normalized contact numbers stored into the Beta-Factor PDB field for easy structure visualization using either VMD 1.9.2 or PyMOL 2.2.2. The best scored poses were further evaluated using molecular dynamics simulations in GROMACS (2016.5-intel-2018-GPU-enabled). GROMACS was run using standard established protocols, but, in short, the GROMOS9643a1 force field was used with a system prepared from the 4FL2 complex with ceftazidime from the best scored Autodock results. The total system charge was neutralized in an explicit water box with 10 Å distance between the protein/ceftazidime complex and the box edge for periodic boundary conditions. Energy minimization was performed to remove interatomic clashes and then NVT ensemble sampling for equilibration was Vcompleted to define the initial conditions of 10 ns MD simulation production runs.

### Quantification and Statistical Analysis

All statistical analysis and details for experiments can be found in the figure legends, the *Methods*, and the *Results*. Microsoft PowerPoint was used to generate the figures. GraphPad Prism was used to generate graphs in 1G-L, 2A-C, 3A-D, 4A, 4C, 5D, 6B-D, S1, S3B, S3D, S3E, S4, S5, and S6. FlowJo was used to generate **Figure 4B**. MATLAB was used to complete KMeans statistical analysis of docking poses. VMD was used to generate **Figures 5A–C, E**, and **S7**. PyMOL was used to generate **Figures 5F, G**, and GRACE was used to generate the graph in **Figure 5H**.

## Results

### Integrin Activation of Syk and Direct Binding of Syk to the Integrin β3 Cytoplasmic Domain

Syk is phosphorylated upon integrin engagement of the ligand (17, 18), which can involve its direct interaction with integrin cytoplasmic domains (20, 21) (**Figure 1A**). When the myelomonocytic cell line THP-1 is plated on the integrin α4β1 substrate VCAM-1, phosphorylation of Syk on multiple tyrosine residues (Tyr323, Tyr352, Tyr 525/526) is rapid and prolonged (**Figure 1B**). Integrin-induced phosphorylation of Syk can be recapitulated with plastic immobilized monoclonal antibodies specific for the integrin β1 subunit (mAb TS2/16), the β2 subunit (mAb 76C3), or the integrin αVβ3 (mAb LM609) (**Figure 1C**). This phosphorylation is independent of the potential interaction of cellular Fc receptors with immobilized goat-anti-mouse capture antibody, as forced non-specific adhesion to GAM-coated surfaces with the use of poly-L-Lysine does not result in Syk phosphorylation (**Figure 1C**). Model peptides of the integrin cytoplasmic domains have demonstrated their direct interactions with intracellular effector molecules, including Syk (39). To develop a binding assay between integrin cytoplasmic domains and Syk, we synthesized integrin β-chain cytoplasmic domains with an N-terminal Gly-Lys-Gly-Gly sequence in which the Lys residue was modified with a biotin-conjugated long-chain (LC) carbon linker (**Figure 1D**). Syk kinase is modular and comprises N-terminal tandem SH2 domains followed by a large kinase domain (**Figure 1E**) (40). The integrin cytoplasmic domain binding site localizes to a region in Syk within the tandem SH2 domains (residues 6-370) (20). Although integrin β1, β2, and β3 cytoplasmic domains can directly interact with Syk, β3 was chosen for assay development because its relative binding affinity is higher than that of the other two integrin cytoplasmic domains (21). We developed an HTS based on the AlphaScreen format because it is a homogeneous system amenable to miniaturization and has been used to screen protein:protein interactions (26–29). A schematic of the assay format (including the null screen) is provided in **Figure 1F**. To first demonstrate binding between the integrin β3 cytoplasmic domain constructs and GST-Syk(6-370), we performed ELISA-based assays in which plastic adsorbed neutravidin was used to capture biotinylated β3 peptide, and GST-Syk(6-370) binding was determined by using anti-GST-HRP conjugated antibodies. GST-Syk(6-370) binding to the integrin β3 cytoplasmic domain was dose-dependent, saturable, and specific (**Figure 1G**), demonstrating that the synthetic peptide was appropriate for further assay development. For AlphaScreen assay development, avidin-coated donor beads were used to capture biotinylated β3 cytoplasmic domain peptide. Anti-GST-conjugated acceptor beads were used to capture GST-Syk(6-370). The binding between the integrin β3 cytoplasmic domain peptide and Syk juxtaposed the donor and acceptor beads, resulting in a fluorescent signal after donor beads are activated at 680 nm. β3 cytoplasmic domain interaction with GST-Syk(6-370) was dose dependent and selective, as no signal was generated when purified GST was conjugated to the acceptor beads (**Figure 1H**). Furthermore, the GST-Syk(6-370) binding curve demonstrated the classic biphasic “hook” effect, whereby excess GST-Syk(6-370) competes in binding to acceptor beads and β3 peptide resulting in a diminished signal. A shortened β3 cytoplasmic domain peptide [β3(sh)] (**Figures 1D, I**) also binds GST-Syk(6-370) (20) (**Supplemental Figure S1**). Deletion of the four C-terminal residues of the integrin β3 cytoplasmic domain decreases Syk binding (20). This peptide was synthesized (β3sh759X, **Figure 1D**) and tested in an ELISA-based format, which demonstrated decreased binding to GST-Syk(6-370) (**Figure 1I**). When tested in the AlphaScreen format, decreased binding was also observed (**Figure 1J**), again demonstrating specificity of the assay. The N-terminal SH2 domain of Syk is sufficient for interaction with integrin cytoplasmic domains (21). As such, GST-Syk(1-99) binds integrin β3 cytoplasmic domain (**Figure 1K**). As a final test of AlphaScreen assay specificity, we removed the GST tag from GST-Syk(6-116) by thrombin cleavage, and we used this purified isolated N-terminal SH2 domain (**Supplemental Figure S2**) as a competitive inhibitor in the AlphaScreen assay. As demonstrated in **Figure 1L**, Syk(1-116) attenuated the signal in a dose-dependent fashion. The AlphaScreen assay is susceptible to false-positive “hits” from compounds that can, for example, quench fluorescent signal or react with singlet oxygen. Thus, a counter-screen was developed to identify false-positive hits (**Figure 1F**, right schematic and **Supplemental Figure S3A**). For this assay, we expressed and purified GST and then modified it with biotin linked through a (PEG)_3_ spacer (Solulink Sulfo ChromaLink Biotin) to a molar substitution ratio of approximately 1. This was calculated based on absorption of the bis-aryl hydrazone in the biotinylation linker domain (**Supplemental Figure S3B**) and GST protein concentration. Integrity of the biotinylated protein was verified by SDS-PAGE (**Supplemental Figure S3C**). Biotinylated GST demonstrated a dose-dependent hook effect, and unmodified GST did not generate a signal (**Supplemental Figure S3D**). Soluble unmodified GST was used as an inhibitor to demonstrate assay specificity (**Supplemental Figure S3E**). The primary β3:GST-Syk(6-370) AlphaScreen and false-positive counter-screen appeared sufficiently tractable to translate into an HTS format.

### HTS Assay Validation and Screen of Prestwick Chemical Library Collection

**Figure 2:**
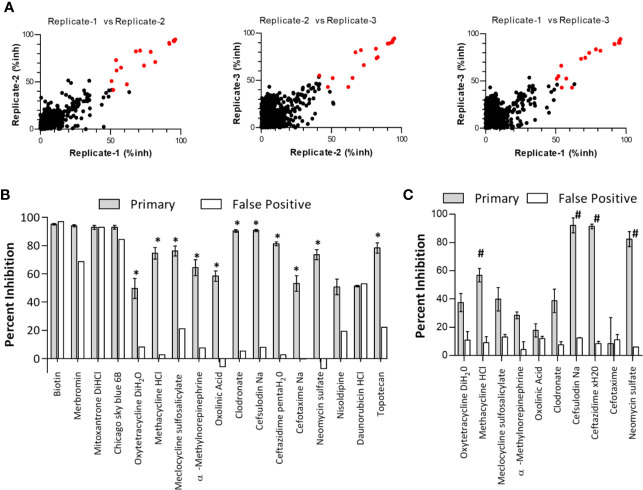
Verification of primary screen “Hit” specificity and activity. **(A)** Primary high throughput screening hits. The primary screen of the Prestwick Compound Collection was performed in triplicate, and scatter plots of combinations of replicates are shown. Negative inhibition was omitted from the plots. Primary hits were identified as compounds with an average of ≥50% inhibition from all three replicates (highlighted as red data points). inh, inhibition. **(B)** From the primary screen, compounds were considered hits if they demonstrated an average inhibition of ≥50% at 30 uM, and a greater than 3-fold difference in activity between the primary screen (gray bars) and the false-positive screen (white bars). Asterisks (*) designate hits carried forward. Data are expressed as the mean percent inhibition ± SEM from three screening runs (note that the secondary false-positive screen was run only once). **(C)** Hits (identified by asterisks in panel B) were reordered from different commercial vendors to verify compound activity by retesting in the primary and false-positive screens at 30 uM. Note that topotecan was not retested due to a lack of solubility. Data are expressed as the mean percent inhibition ± SEM from three experiments (note that ceftazidime xH20 and cefotaxime false-positive screens were performed only twice). ^#^denotes compounds fulfilling the original hit criteria.

In a checkerboard dose-response analysis of biotinylated β3 peptide and GST-Syk(6-370), concentrations of 100 nM peptide and 0.3 nM GST-Syk(6-370) gave the highest signal:background ratios (between 10 and 20 fold) for the screen. When these analyte concentrations were tested in an automated HTS system, the signal:background ratios ranged from 6 to 10 fold. In the automated systems, we tested adding reagents in a different order. A maximal signal was generated when reagents were added in a sequential manner starting with the addition of GST-Syk(6-370) to anti-GST acceptor beads (with a 1-h incubation at room temperature), followed by the addition of biotinylated β3 peptide (1-h incubation at room temperature) and then avidin-coated donor beads. Kinetic tests indicated that the maximal signal was completely generated by 4 h after the final addition of donor beads. DMSO sensitivity tests indicated the assay could withstand 1% DMSO with no effect on the maximum signals generated. The pilot HTS was to be performed in the presence of 0.25% DMSO; therefore, this finding was within the limits of the assay, and a pilot screen was initiated. To determine assay reproducibility, we screened the 1,280-compound Prestwick Chemical Library Collection on three separate occasions (**Figure 2A**). The following is a summary of the screening metrics from the primary screen: average maximum signal = 30,908; average SD of maximum signal = 2,052; average S/B ratio = 5.6; and the average Z’-score = 0.7. A false-positive counter-screen was performed once, and the metrics were as follows: average maximum signal = 44,456; average SD of maximum signal = 2,134; average S/B ratio = 29; and the average Z’-score = 0.8. The final concentration of compounds tested was 25 uM. A scatter plot of the assay results comparing all repeat combinations is presented in **Figure 2A**. From the primary screen, a total of 17 compounds demonstrated an average of ≥50% inhibition of fluorescent signal (**Figure 2B**). However, several compounds also demonstrated inhibition in the false-positive counter screen (**Figure 2B**, white bars). Using a hit criteria of greater than 50% inhibition of fluorescent signal in the primary screen and a greater than 3-fold difference in inhibition over the false-positive counter screen, we identified 11 preliminary hits (**Figure 2B**, indicated by asterisks). The hits from the primary and secondary false-positive screens were next obtained from a separate commercial source and verified for activity. Most of the re-sourced compounds demonstrated some level of inhibitory activity; however, only four of these fulfilled the original hit criteria as outlined above (**Figure 2C**, indicated by the number sign). This finding represents a true hit rate of 0.3 percent from the Prestwick library collection. The hits that were identified in the AlphaScreen were the following antibiotics: methacycline, cefsulodin, ceftazidime, and neomycin sulfate. Based on the success of this screen, we also screened the Maybridge Hitfinder library of 14,400 compounds. Although 32 compounds were identified as hits in the primary screen, none of the re-sourced compounds retained the 3-fold difference between primary and null screens (**Supplemental Figure S4**).

### Hit Potency and Orthogonal Screens

**Figure 3:**
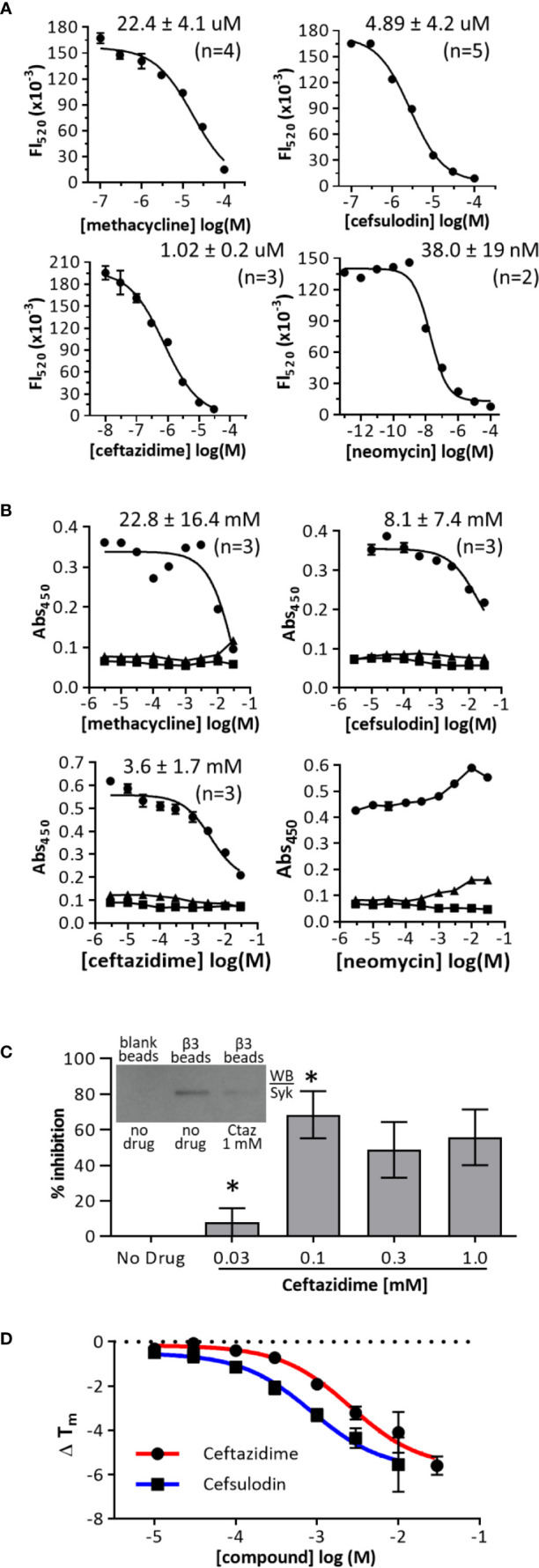
Dose-response curves in primary and orthogonal screens. **(A)** AlphaScreen dose-response curves. Biotinylated-b3(sh) peptide was used at a final concentration of 30 nM, and GST-Syk(6-370) was used at a final concentration of 0.3 nM. Data are expressed as mean fluorescence (Fl_520_) ± range from duplicates, and one representative experiment of “n” performed is shown. **(B)** Orthogonal ELISA-based dose-response assays. Biotinylated-b3(long) peptide was used at a coating concentration of 1 µg/ml, and GST-Syk(6-370) was used at a final concentration of 0.3 nM (○); GST only (▪); no β3 (black Δ). Data are expressed as mean abs_450_ ± SEM from triplicate determinations, and one of three representative experiments is shown. **(C)** Ceftazidime inhibition of β3 peptide binding to Syk from THP-1 whole cell lysate. THP-1 cell lysate (100 ug) was treated with or without ceftazidime (Ctaz) before incubation with β3-coated avidin-agarose beads, and bound Syk was measured by western blot (example shown in inset). Blank beads served as a negative control. Equal β3 peptide loading was verified by probing blots with avidin-HRP (not shown). Data were quantified as mean percent inhibition ± SEM (n = 3). *p < 0.01 (Tukey *post-hoc* comparison) **(D)** Thermal denaturation curves of GST-Syk(6-370). GST-Syk(6-370) was used at a concentration of 2 µM, and denaturation was measured *via* SYPRO Orange fluorescence.

To determine the IC_50_ of the hit compounds, we performed dose-response analyses (**Figure 3A**). In the AlphaScreen assay, the most potent drug was neomycin sulfate (IC_50_ = 38.0 ± 19 nM), followed by ceftazidime (IC_50_ = 1.02 ± 0.2 µM), cefsulodin (IC_50_ = 4.9 ± 4.2 µM), and methacycline (IC_50_ = 22.4 ± 4.1 µM). The Hill coefficients of dose-response curves were close to 1.0. As an orthogonal screen, we performed ELISA-based assays with bβ3(fl) and GST-Syk(6-370). Under these assay conditions, methacycline was not soluble at the highest doses tested, and neomycin sulfate did not demonstrate inhibitory activity. Cefsulodin and ceftazidime remained active with a similar rank-order of activity; however, they were less potent in this assay format than in the AlphaScreen format (**Figure 3B**). Ceftazidime (the most potent of the two remaining hits) was further tested in assays of whole cell lysates to verify the antagonism of the binding of wild-type full-length Syk to integrin cytoplasmic domains. Neutravidin-coated agarose beads were bound with bβ3(fl) and incubated with THP-1 whole cell lysate, with or without ceftazidime. Bead-bound Syk was detected by western blot. Ceftazidime inhibited wild-type Syk interaction with integrin β3 cytoplasmic domains (**Figure 3C**). We performed thermal denaturation experiments to demonstrate interactions between hits (ceftazidime and cefsulodin) and GST-free Syk(6-370). Syk(6-370) melting temperatures (T_m_) were measured with and without compound. Cefsulodin and ceftazidime decreased the T_m_ of Syk(6-370) in a dose-dependent fashion (**Figure 3D**).

### Effects of Ceftazidime on Integrin Signaling *via* Syk

**Figure 4:**
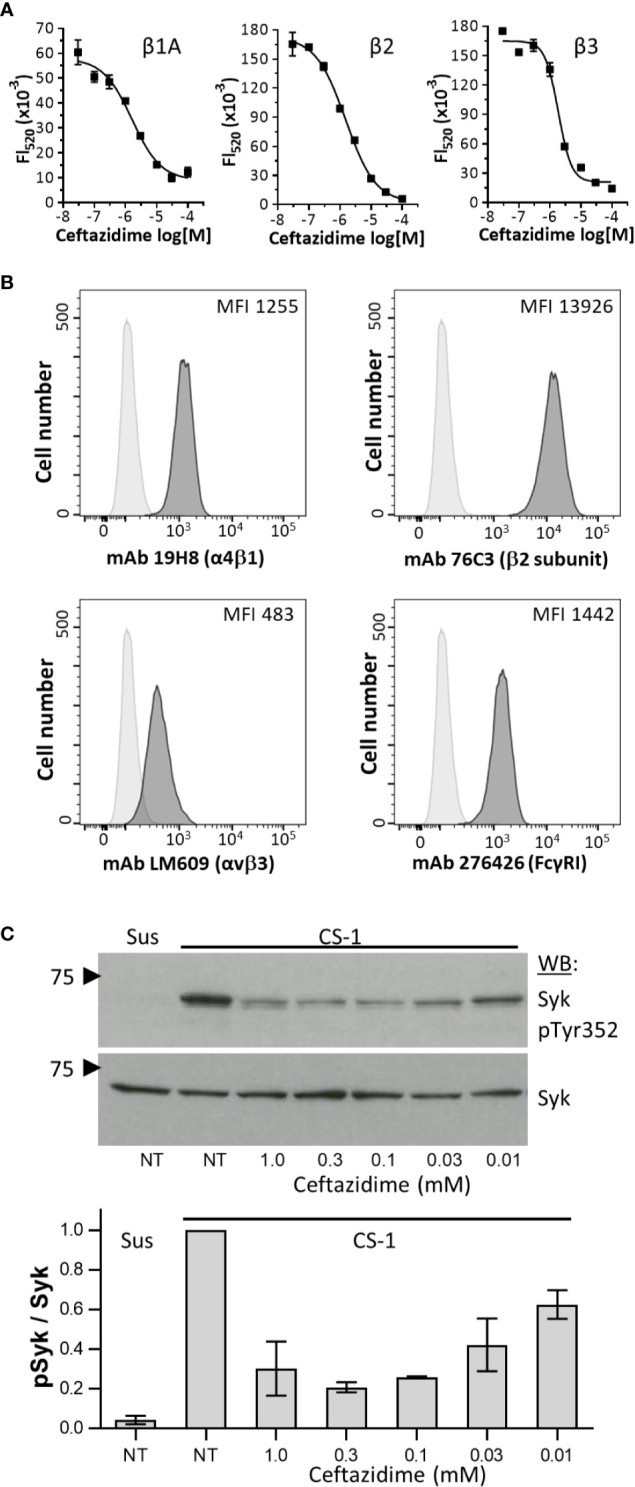
Ceftazidime inhibition of integrin signaling. **(A)** Dose-response curves comparing ceftazidime antagonist activity against integrin β1A, β2, and β3 cytoplasmic domains binding to Syk as measured in AlphaScreen assays. For all dose-response curves, biotinylated-β chain peptide was used at a final concentration of 30 nM, and GST-Syk(6-370) was used at a final concentration of 0.3 nM. Data are expressed as mean fluorescence (Fl_520_) ± the range from duplicate determinations. One of three experiments is shown. **(B)** Flow cytometric analysis of integrin α4β1, β2 subunit, αVβ3, and FcγRI (CD64) on THP-1 cells. Isotype control staining is shown as light gray histograms. Specific integrin staining is shown as darker gray histograms. One of three representative experiments is shown. **(C)** Dose-dependent effects of ceftazidime on integrin-dependent phosphorylation of Syk. Ceftazidime was loaded into THP-1 by glycerol shock. Control cells (NT) underwent glycerol shock but in the absence of drug. THP-1 cells were either maintained in suspension (Sus) or plated on plastic immobilized CS-1 conjugated to bovine serum albumin. After incubation at 37°C, whole cell lysates were separated by SDS-PAGE, probed for phosphorylated Syk (pY352, mAb 65E4), stripped, and then re-probed with monoclonal antibody 4D10 to determine total Syk levels. One representative blot is shown (top), along with quantification of three separate experiments normalized to maximum Syk phosphorylation (bottom). Results are expressed as the mean ± SEM.

Integrins of the β1, β2, and β3 families can signal through the tyrosine kinase Syk (**Figure 1C**) (17, 18, 41). To determine whether ceftazidime was a selective inhibitor of integrin cytoplasmic domain interactions with Syk, we performed AlphaScreen assays using β1, β2, and β3. Ceftazidime was equally active against all three integrins (1.62 ± 0.41 µM, 1.53 ± 0.21 µM, and 1.87 ± 0.2 μM, respectively) (**Figure 4A**). We used the THP-1 cell line in experiments designed to determine whether antagonists of the integrin β3 cytoplasmic domain interaction with Syk would prevent integrin signaling *via* Syk. Integrin expression on THP-1 cells was analyzed by flow cytometry to determine expression levels of integrins α4β1, β2, and αvβ3, and the immune response receptor FcγRI; we wanted to identify integrins that were expressed at similar levels as FcγRI because our goal was to determine if antagonists of integrin:Syk interactions would also affect the canonical activation of Syk that occurs *via* interaction with dually phosphorylated ITAMs (pITAMs) found within transmembrane adaptor molecules associated with immune response receptors such as FcγRI (42). Of the three integrins tested, integrin α4β1 was expressed at similar levels as FcRγI (**Figure 4B**). Because ceftazidime is equally active against all three integrins *in vitro* (**Figure 4A**), we examined the effects of ceftazidime on integrin α4β1 signaling through Syk. The results were negative in initial experiments performed by simply adding ceftazidime to THP-1 cells and examining α4β1 signaling through Syk. We hypothesized this was due to a low accumulation of ceftazidime within cells, which led to the development of a facile approach of loading THP-1 cells with the drug by using glycerol shock. This method was validated with a fluorescent probe, sulfo-Cy5 (**Supplemental Figure S5**), which demonstrated accumulation of the dye in THP-1 on increasing concentrations of glycerol. In this method, THP-1 cells were loaded with ceftazidime (at indicated doses) or vehicle (DMSO) control, rested for 10 min in serum-free medium (RPMI-1640) containing drug or vehicle control, and then plated on the α4β1 integrin substrate CS-1. Cells kept in suspension demonstrated little phosphorylation of Syk Tyr352 (**Figure 4C**). However, when cells adhered to CS-1, prominent tyrosine phosphorylation was observed (**Figure 4C**). In the presence of ceftazidime, α4β1-induced phosphorylation of Syk was inhibited (**Figure 4C**). This was not due to ceftazidime inhibition of THP-1 cell adhesion to CS-1 (**Supplemental Figure S6**).

### Modeling Ceftazidime Binding to Syk Tandem SH2 Domains

**Figure 5:**
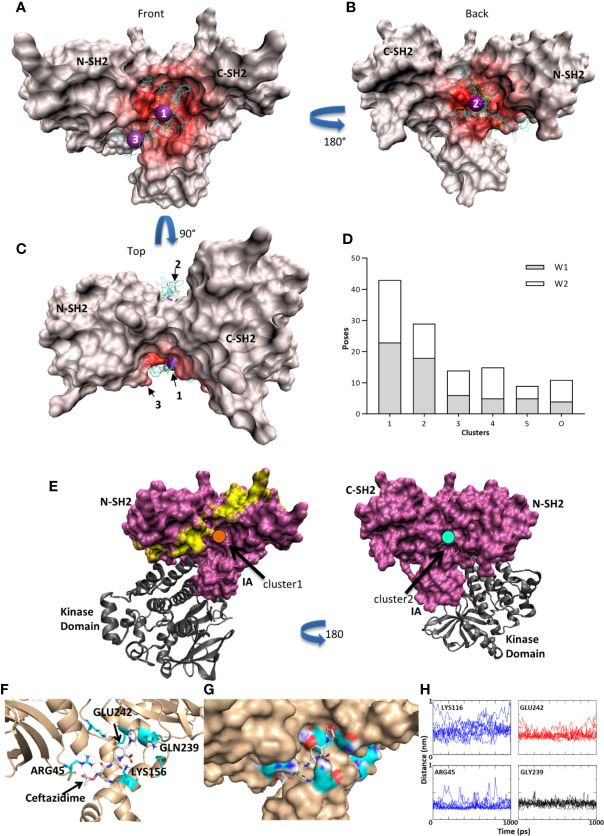
Syk kinase/ceftazidime binding. **(A)** Red heat map indicates the number of contacts between Syk and the docked poses. A few typical poses are shown for illustration purposes. Purple spheres are the centroid of clusters 1 and 3 in the front view. **(B)** Heat map of contacts on the rear view and purple sphere at the centroid of cluster 2. **(C)** Rotation of 90 degrees to the top view. **(D)** Histogram of clusters. Clusters 1 and 2 have the largest populations and are characterized by fitting into the pocket at the tandem SH2 domain and interdomain interface on the front and back. Window 1 (W1) is a large unbiased search window and window 2 (W2) is a smaller, focused window to increase sampling in the pockets (see **Supplemental Figure S7**). **(E)** Cluster 1 (orange; left panel) is adjacent to the residues that would interact when a pITAM peptide (yellow surface) is bound but may allow for co-binding. Cluster 2 (cyan; right panel) is on the back face and thus interacts with neither pITAM binding areas nor Syk catalytic domain binding interfaces. **(F)** Molecular dynamics simulations in GROMACS. A typical trajectory frame during MD simulations with residues GLN239 and LYS156 making stabilizing interactions. ARG45 and GLU242 also contribute. **(G)** The same trajectory frame as shown in **(F)** illustrating one possible binding mode in the groove between the N-term SH2 domain and the IA domain. **(H)** Distances between ceftazidime and LYS116, GLU242, ARG45, and GLN239 during one representative 10 ns trajectory (1 ns is displayed).

Ceftazidime was docked (Autodock Vina 1.1.2) (34, 35) onto the Syk tandem SH2 domains (PDB 4FL2) (36). Cluster analysis indicated several possible binding sites for ceftazadime within the tandem SH2 domains of SYK. **Figures 5A–C** show three clusters from both the large (W1) and focused (W2) search windows that span the entire tandem SH2 domain (**Supplemental Figure S7**). The heat maps identify regions in the intersection between the N-terminal and C-terminal SH2 domains, along with the interdomain A region. The K-means analysis of docking poses shows significant preference for clusters 1 and 2. Cluster 1, located at (−10.9, −5.3, 30.0) Å in the PDB frame, has 43 members that average 3.9 +/− 3.4 Å within that cluster centroid (**Figure 5D**, **Figure 5A** purple sphere 1) and is adjacent but not in direct competition with the residues known to interact between pITAM peptides and the tandem SH2 domain (**Figure 5E**; yellow shaded surface) (43). Both cluster 1 and cluster 2 interact with the tandem SH2 domain in areas that are not dominated by interactions with the SYK catalytic domain (**Figure 5E**) (36). Molecular dynamics simulations (10 ns each) of the ceftazidime and Syk complex further reinforced that cluster 1 is a putative binding mode with interactions between ceftazidime and GLN239 (2.98 +/− 0.48 Å) and ARG45 (2.57− 0.7 Å) on Syk in the interdomain A region. Ceftazidime also interacted with GLU242 (3.64 +/− 0.73 Å) and was in the proximity of LYS116 (4.5 +/− 1.3 Å) (**Figures 5F–H**).

### Ceftazidime and Selective Inhibition of Integrin Signaling *via* Syk

**Figure 6:**
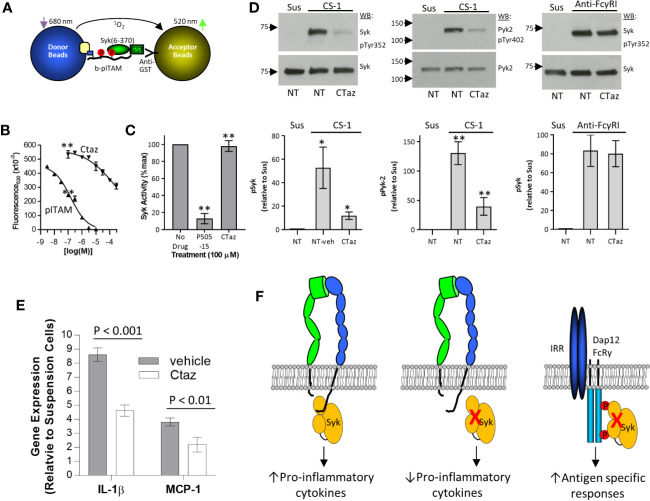
Selective inhibition of integrin signaling by ceftazidime. **(A)** Schematic illustration of the pITAM-Syk AlphaScreen used to test antagonist selectivity. **(B)** Comparison between ceftazidime (Ctaz) and pITAM peptide inhibition of pITAM-Syk binding in the AlphaScreen assay described in **(A)**. Biotinylated pITAM peptide was used at 10 nM, and GST-Syk(6-370) was used at a final concentration of 1 nM. Data are expressed as mean ± range from duplicate determinations. One of four representative assays is shown. **(C)** Effect of ceftazidime on Syk kinase activity. Ceftazidime (Ctaz) or Syk inhibitor P505-15 was used at 100 uM. Kinase activity is expressed as percent of maximum signal. Data are expressed as mean ± SEM (n = 3). **p < 0.01 **(D)** Selective effects of ceftazidime on integrin signaling. Ceftazidime (Ctaz; 100 uM)-loaded THP-1 or mock-loaded control cells (NT) were either maintained in suspension (Sus) or incubated on immobilized CS-1 (left and center panels) or anti-FcγRI monoclonal antibody 276426 (right panel). Phosphorylation of Syk (left and right panel) and Pyk-2 (center panel) was analyzed by immunoprecipitation and western blot. One representative blot is shown. Quantification is shown below (left panel, n = 5; center panel, n = 3; right panel, n = 3), normalized to phosphorylation in suspension. Results are expressed as the mean ± SEM. ANOVA Tukey *post-hoc* analysis; *p < 0.05; **p < 0.01. **(E)** Similar to **(D)**, cells were loaded with ceftazidime (Ctaz; 100 uM) or mock-loaded and kept in suspension or plated on CS-1. Expression of interleukin (IL)-1β and monocyte chemoattractant protein (MCP)-1 genes was examined by quantitative real-time polymerase chain reaction. Data are expressed as expression levels relative to control cells kept in suspension. Results are shown as the mean± SD. P-values were calculated by the Student *t* test. **(F)** Cartoon representation of integrin and immune response receptor signaling *via* Syk.

The modeling results suggest ceftazidime binding to Syk tandem SH2 domains would have limited effects on the interaction between pITAM peptides and Syk and on Syk’s catalytic activity. In AlphaScreen assays that measured the binding between pITAM and the Syk tandem SH2 domains (**Figure 6A**), ceftazidime had limited activity (IC_50_ > 300 uM), although free pITAM peptide completely inhibited this interaction (IC_50_ = 97.1 ± 29.2 nM; n = 3) (**Figure 6B**). As expected, ceftazidime had no effect on the catalytic function of full-length Syk (**Figure 6C**) despite testing at 100-fold higher concentration than the AlphaScreen IC_50_. On the basis of these results and modeling, we hypothesized that ceftazidime may inhibit integrin signaling *via* Syk but leave canonical FcγRI signaling *via* Syk intact. THP-1 cells were loaded with ceftazidime (100 µM) and plated on α4β1 substrate CS-1 or anti-FcγRI mAb (to crosslink this receptor). Integrin α4β1 induced phosphorylation of Syk, and proline-enriched tyrosine kinase-2 (Pyk-2), a kinase that is downstream of Syk (44), was inhibited by incorporation of ceftazidime (**Figure 6D**). Integrin signaling in THP-1 cells resulted in upregulation of pro-inflammatory cytokines such as IL-1β and MCP-1 (**Figure 6E**). Similar to phosphorylation of Syk, adhesion-dependent induction of the expression of IL-1β and MCP-1 was significantly inhibited by incorporation of ceftazidime (**Figure 6E**). However, when FcγRI was crosslinked on the surface of ceftazidime-loaded cells, tyrosine phosphorylation of Syk was not inhibited (**Figure 6D**).

## Discussion

Integrin signaling through the non-receptor tyrosine kinase Syk is due to direct interactions between Syk and integrin β-chain cytoplasmic domains (20). In this report, we have identified a β-lactam–containing cephalosporin antibiotic ceftazidime that inhibits integrin signaling through Syk without inhibiting canonical activation of this kinase by immune response receptors such as FcγRI. This is the first description of the selective pharmacological inhibition of integrin signaling through Syk. Our findings suggest a novel approach to the discovery and development of therapeutic agents targeting inflammation and autoimmunity.

From a screen of over 1,280 known drugs and 14,400 small molecule compounds (from the Prestwick Chemical and Maybridge HitFinder libraries), we identified cephalosporin antibiotics, ceftazidime, and cefsulodin, as antagonists of integrin β-chain cytoplasmic domain interactions with the tandem SH2 domains of Syk. In AlphaScreen assays, ceftazidime and cefsulodin had an IC_50_ of 1.0 ± 0.2 × 10^−6^ (M) and 4.2 ± 4.0 × 10^−6^ (M), respectively. Activity was verified in orthogonal ELISA-based assays. The IC_50_ was 3.6 ± 1.7 × 10^−3^ (M) and 8.1 ± 7.6 × 10^−3^ (M) for ceftazidime and cefsulodin, respectively. Compounds were less active in the ELISA-based orthogonal assays (**Figure 3B**), which has been previously reported in a comparison of AlphaScreen and ELISA-based assay formats (45). Ceftazidime inhibited endogenous Syk (from THP-1 cell lysate) binding to β3 cytoplasmic domain peptides immobilized on sepharose beads (**Figure 3C**). Thermal denaturation curves of the tandem SH2 domains of Syk in the presence of ceftazidime indicated a destabilizing effect of compound binding (**Figure 3D**) (46), which suggests that ceftazidime acts through binding the tandem SH2 domains of Syk and not the integrin cytoplasmic domains. When loaded into THP-1 cells, ceftazidime inhibited integrin-dependent phosphorylation of Syk (**Figure 4C**) and adhesion-dependent expression of the inflammatory cytokines IL-1β and MCP-1 (**Figure 6E**), independent of the effects on cell adhesion (**Supplemental Figure S6**). In summary, ceftazidime inhibited integrin β-chain cytoplasmic domain interactions with Syk in four different assay formats, including cell-based integrin signaling assays.

There is debate as to whether integrin signaling *via* Syk requires Syk SH2 domain interactions with phosphorylated ITAMs, or whether integrins signal *via* Syk in an ITAM-independent manner. Initial studies in heterologous expression systems demonstrated that integrin signaling through Syk was ITAM independent (20, 21, 47). Selective inhibition of integrin-dependent phosphorylation of Syk with ceftazidime, while leaving FcγRI-induced phosphorylation of Syk intact, is consistent with this model. Also supporting this model are observations of αllbβ3 signaling in platelets from *Syk^R41Afl/fl;PF4-Cre^* mice that demonstrate normal αIIbβ3-dependent phosphorylation of Syk*^R41A^* despite abrogated GPVI and CLEC-2 signaling, which requires an intact phosphotyrosine binding N-SH2 domain (48). *In vitro* studies (22) also support ITAM-independent integrin coupling to Syk; these investigators found binding of Syk to integrin cytoplasmic domains presented in high density (to model integrin clustering) resulted in Syk transphosphorylation independent of ITAM interactions. However, integrin signaling in neutrophils and macrophage was shown to be ITAM-dependent in studies in primary cells in which pITAM signaling of Syk was abrogated by retroviral reconstitution of Syk^−/−^ cells with Syk containing non-functional SH2 domains or when pITAM signaling adaptors such as DAP12 or FcRγ were knocked out (10, 49). It is unclear whether this represents differences in integrin signaling *via* Syk in disparate cell types or technical differences in the model systems tested. Nevertheless, the mechanisms by which integrins couple with Syk can clearly be both ITAM-independent and ITAM-dependent, and as described by Mocsai et al. (23), these mechanisms need not be mutually exclusive and most likely occur together as integrin co-signaling with a variety of cell surface receptors appears to converge on Syk and Syk family kinases. By demonstrating that ceftazidime can selectively inhibit integrin signaling through Syk while not altering the ability of FcRs to signal *via* Syk, we provide further evidence of the independent regulation of this kinase by these two different receptor systems.

Syk activation occurs through direct interaction with phosphorylated immune-receptor tyrosine-based activation motifs (50) or through integrin clustering that results in transphosphorylation on tyrosine residues and activation of catalytic function (22). The interaction of Syk with pITAM containing immune response receptors or signaling adaptor molecules is well defined, as is the resultant conformational changes that lead to activation of Syk kinase activity (36, 43). However, the interaction between integrin cytoplasmic domains and Syk is less well defined. Although the interaction occurs within the tandem SH2 domains of Syk, integrin cytoplasmic domains and pITAMS do not compete for binding to Syk (20, 22) and are in fact additive when used in combination to activate this kinase (22), which suggests independent modes of binding. Modeling of the ceftazidime binding pockets in the Syk tandem SH2 domains identified 2 major interaction clusters that were outside of regions important for interaction between pITAM peptides and Syk (**Figure 5**). These appeared to be at an interface between the SH2 domains and interdomain A region. This mode of binding is consistent with the lack of effect of ceftazidime on Syk kinase activity (**Figure 6C**), the relatively poor antagonist activity of ceftazidime against pITAM:Syk interactions (**Figure 6B**), and the lack of effect of ceftazidime on FcRγ1 signaling through Syk (**Figure 6D**). Further high-resolution structural and biochemical studies are necessary to definitively identify a mode of binding of antagonists such as cefsulodin and ceftazidime to the Syk tandem SH2 domains (including potential covalent modification), along with integrin β-chain cytoplasmic domain interactions with the Syk tandem SH2 domains. These studies may also aid in identifying specific regions in the tandem SH2 domains of Syk that specifically interact with integrin β-chain cytoplasmic domains.

A limitation of this work is that the screens performed were cell free, which precluded identifying small molecule compounds that could both inhibit integrin cytoplasmic domain interactions with Syk and accumulate intracellularly. As such, the compounds identified had to be loaded into THP-1 cells by glycerol shock to demonstrate their effects on integrin signaling in cells (**Figure 6**). Studies are currently ongoing to identify small molecule compounds that are predicted to be membrane permeable and that models would predict to have a similar mode of binding to the Syk tandem SH2 domains as the β-lactam antibiotics identified here. Identifying such compounds will be useful as chemical probes to ascertain the importance of integrin signaling *via* Syk in inflammatory and autoimmune disease models.

Currently, all drugs approved for human use that target integrin cell adhesion molecules are antagonists that prevent cell adhesion. These include the αIIbβ3 targeting drugs abciximab (51, 52), tirofiban (53, 54), and eftifibatide (55); the α4β1 and α4β7 targeting humanized mAb natalizumab (56); the α4β7 humanized mAb vedolizumab (57, 58); and the αLβ2 drugs efalizumab (59) (withdrawn from the market due to safety concerns) (60) and lifategrast (61). These first-generation integrin antagonists all target integrin ectodomains and thus prevent both cell adhesion and integrin signal transduction. Similarly, inhibitors of Syk that target its kinase domain, such as fostamatinib (approved for use in immune thrombocytopenia), lack selectivity (62). Thus, current inhibitors of Syk have off-target selectivity issues and cannot differentially inhibit signaling *via* one upstream receptor over the other. As demonstrated here and elsewhere (63, 64), identifying antagonists of integrin cytoplasmic domain interactions with intracellular signaling effector molecules or adaptor proteins may lead to selective targeting of integrin signaling pathways that contribute to disease pathogenesis, while limiting the effects on cell adhesion, thereby leaving potentially beneficial integrin signaling pathways intact.

## Data Availability Statement

The raw data supporting the conclusions of this article will be made available by the authors, without undue reservation.

## Author Contributions

Conceptualization, DW and CS. Methodology, DW, CS, DB, PV, and JC. Validation, AK, GB, and NN. Formal Analysis, JC, AA, SA, HP. Investigation, DB, AK, NN, GB, AC, CG, HP, SG, and SL. Data Curation, CS. Writing—Original Draft, DW, DB, JC, and AC. Writing—Review and Editing, DW, DB, JC, AC, AK, CS, PV. Visualization, DW, AC, and JC. Supervision, DW, PV, JC, and CS. Funding Acquisition, DW, CS, and JC. All authors contributed to the article and approved the submitted version.

## Funding

Grant support: R01AI095575 (DW). Microsoft CRM:0740135 and CACDS SeFAC:63445-B5006 provided support for computational resources (JC).

## Conflict of Interest

The authors declare that the research was conducted in the absence of any commercial or financial relationships that could be construed as a potential conflict of interest.
