# Supplementary material for: Identification of Inhibitors of Integrin Cytoplasmic Domain Interactions With Syk
Source: Front Immunol. 2021 Jan 8;11:575085. doi: 10.3389/fimmu.2020.575085 (PMC7819857; doi:10.3389/fimmu.2020.575085)
Supplement: Supplementary file 1 [file DataSheet_1.docx]

Supplementary Material

# Supplementary Figure Legends

**Supplemental Figure 1**. ELISA-based assay of full-length and shortened integrin β3 cytoplasmic domain binding to GST-Syk(6-370).

**Supplemental Figure 2.** Purification of GST free Syk(1-116). Purified GST-Syk(1-116) was incubated at room temperature with 10 ul (packed) thrombin agarose beads (Sigma), and samples were collected at indicated time points to check for free GST and Syk(1-116). Digestion approached completion at 24 hours (left panel). Scale-up digestions of GST-Syk(1-116) were performed for 24 hours in 0.2 ml, at 4^0^C with thrombin agarose beads (20 ul, packed). After 24 hours, free GST was removed from the supernatant by incubating overnight at 4^0^C with glutathione sepharose beads (GE Healthcare). Right panel shows before (-) and after (+) thrombin treatment in addition to removal of GST, showing purified Syk (1-116), which is just above the dye front at slightly higher than the 10 kDa molecular weight marker.

**Supplemental Figure 3**. Secondary false-positive screen. **(A)** Schematic showing the secondary false-positive screen. The same assay format is utilized except no β3 cytoplasmic domain is added, and GST-Syk(6-370) is replaced with biotinylated GST, which has been titrated to obtain a similar maximum signal as the primary screen. **(B)** Calculation of the molar substitution ratio (MSR) of biotin-LC-modified GST. GST was expressed in BL21 cells and purified on a GSH-column. It was modified with biotin linked through a (PEG)_3_ spacer that contains an internal bis aryl hydrazone (λ_max_ 354 nm). The MSR was calculated to be approximately 1. **(C)** Biotin-LC-modified GST. Biotin-LC-GST (b-LC-GST) was compared to unmodified GST by SDS-PAGE. **(D)** Dose-dependent generation of AlphaScreen signal with biotinylated GST. Biotinylated GST or unmodified GST was incubated with acceptor beads. After a 1-hour incubation, streptavidin-conjugated donor beads were added. After a 1-hour incubation at room temperature, assay plates were read on a plate reader. The dose-response of biotinylated-GST demonstrates the typical “hook” effect of AlphaScreen assays. Data are presented as mean ± the range from duplicate determinations. One of three representative experiments is shown. **(E)** Competition of biotinylated-GST false-positive screen with free GST. Increasing concentrations of un-modified GST were used to compete with anti-GST acceptor beads. Data are presented as mean ± the range from duplicate determinations. One of two representative experiments is shown.

**Supplemental Figure 4**. Maybridge Library Screen. **(A)** Representative primary screen (1 of 2 performed). **(B)** Re-sourced compounds representing various structure classes of hits from the primary screen.

**Supplemental Figure 5**. Glycerol loading of compound into cells. Cells were subjected to glycerol shock as described in the Methods. A water-soluble sulfo-Cy5 dye was used to indicate incorporation of the compound. After glycerol shock (using indicated concentrations of glycerol), dye incorporation was analyzed by flow cytometry. In the absence of glycerol, no significant accumulation of dye was observed in cells (not shown). One of three representative experiments is shown.

**Supplemental Figure 6.** Effects of ceftazidime on THP-1 adhesion to CS-1. Cells were loaded with ceftazidime by glycerol shock. After loading, cells were washed with media and loaded with calcein-AM. CS-1–conjugated bovine serum albumin was immobilized in 96-well plates (5 ug/ml), and adhesion assays were performed as previously described (1). Data are expressed as average fluorescence ± standard deviation from triplicates. One of two representative experiments is shown. The green line represents cell adhesion in the absence of ceftazidime.

**Supplemental Figure 7**. SYK kinase/ceftazidime binding search windows. The large unbiased search window (W1) was centered at (-11.6,3.6,34.0) Å and sized (90,80,80) Å, and the more focused window (W2) was centered also at (-11.6,3.6,34.0) Å and sized (80,70,70) Å to enrich numerical sampling.

## Supplemental Table 1. Key Resources

| REAGENT or RESOURCE | SOURCE | IDENTIFIER |
| --- | --- | --- |
| **Antibodies** | | |
| Mouse mAb anti-human FcγRI (CD64) | R&D Systems | Cat# 12571; clone 276426;  RRID: AB_2262693 |
| Mouse mAb anti-human β2 integrin clone 76C3 | Dr. Brad McIntyre UTMDACC | N/A |
| Mouse mAb anti-human integrin α4β1 clone 19H8 | Dr. Brad McIntyre; McIntyre, BW et al., 1997 | N/A |
| Mouse mAb anti-human integrin β1 | ATCC (hybridoma) | Cat# HB-243; clone TS2/16 |
| Mouse mAb anti-human integrin αvβ3 | EMD Millipore | Cat# MAB1976; clone LM609;  RRID: AB_2296419 |
| Mouse mAb anti-human Syk | Santa Cruz | Cat# sc-1240; clone 4D10;  RRID: AB_628308 |
| Rabbit mAb anti-phospho Syk-Tyr352 | Cell Signaling Technologies | Cat# 2717; clone 65E4;  RRID: AB_2218658 |
| Mouse mAb anti-phospho Tyr | Upstate Biotechnology-Millipore | Cat# 05-321; clone 4G10;  RRID: AB_309678 |
| Rabbit anti-phospho Syk-Tyr323 | Cell Signaling Technologies | Cat# 2715;  RRID: AB_10828096 |
| Rabbit mAb anti-phospho Syk-Tyr525/526 | Cell Signaling Technologies | Cat# 2710; clone C87C1 |
| Rabbit recombinant mAb anti-phospho Pyk2-Tyr402 | Invitrogen-Life Technologies | Cat# 700632; Clone 17H4L19;  RRID: AB_2532332 |
| Goat anti-mouse IgG HRP conjugate | Southern Biotechnology | Cat# 1030-05;  RRID: AB_2619742 |
| Goat anti-rabbit IgG HRP conjugate | Southern Biotechnology | Cat# 4030-05;  RRID: AB_2687483 |
| Rabbit anti-Pyk2 serum | Upstate Biotechnology-Millipore | Cat# 05-488; clone 74;  RRID: AB_11212673 |
| Rabbit polyclonal anti-GST HRP conjugate clone Z-5 | Santa Cruz | Cat# sc-459 clone Z-5 replaced by sc-138 clone B-14;  RRID: AB_631586 |
| **Bacterial and Virus Strains** | | |
| BL21 (DE3) | Novagen-Millipore | cat# 69450-3 |
| **Chemicals, Peptides, and Recombinant Proteins** | | |
| CS-1-BSA conjugate | New England Peptides | CDELPQLVTLPHPNLHGPEILDVPST |
| GST-Syk 1-116 | This paper | n/a |
| bGST | This paper | n/a |
| GST-Syk 1-99 | This paper | n/a |
| Syk 1-116 | This paper | n/a |
| GST-Syk 1-163 | This paper | n/a |
| GST-Syk 6-370 | Shiue L., et al. (1995) (2) | n/a |
| GST-Syk 165-268 | This paper | n/a |
| biotinylated β1A | New England Peptides | G(K-LC-Biotin)GG-KLLMIIHDRREFAKFEKEKMNAKWDTGENPIYKSAVTTVVNPKYEGK |
| biotinylated β2 | New England Peptides | G(K-LC-Biotin)GG-KALIHLSDLREYRRFEKEKLKSQWNND-NPLFKSATTTVMNPKFAES |
| biotinylated β3 (fl) | New England Peptides | G(K-LC-Biotin) GG-KLLITIHDRKEFAKFEEERARAKWDTANNPLYKEATSTFTNITYRGT |
| biotinylated β3-short (sh) | New England Peptides | G(K-LC-Biotin)GG-RARAKWDTANNPLYKEATSTFTNITYRGT |
| biotinylated β3-(sh759X) | New England Peptides | G(K-LC-Biotin)GG-RARAKWDTANNPLYKEATSTFTNIT |
| VCAM-1-mIgG2a | Woodside, D.G. et al. (1) | n/a |
| **Critical Commercial Assays** | | |
| Thrombin CleanCleave Kit | Sigma | cat# SLBF0950 |
| Solulink Sulfo ChromaLink Biotin | Solulink | cat# B-1007 |
| AlphaScreen GST Detection Kit | Perkin Elmer | cat# 6760603 |
| 1-Step Ultra TMB Elisa | Thermo Scientific | cat# 34028 |
| SYPRO Orange | Thermo Scientific | cat# S6650 |
| SuperSignal West Pico Plus Chemiluminescent Substrate | Thermo Scientific | cat# 34580 |
| **Deposited Data** | | |
| PDB | <https://www.rcsb.org/structure/4FL2> | 4FL2 |
| **Experimental Models: Cell Lines** | | |
| THP-1-human monocyte (gender male) | ATCC | cat# TIB-202;  RRID:CVCL_0006 |
| **Oligonucleotides** | | |
| forward primer for Syk 1-163 5’-tatggcgaattcatggccagcagcggcatgcct-3’ | This paper | n/a |
| reverse primer for Syk 1-163 5’-tatgccctcgagtcaatgggctgtggtagcgatcag-3’ | This paper | n/a |
| forward primer for Syk 1-99 5’-CAGGAGTCTGATGGCTAGGTCTGCCTCCTCAAG-3’ | This paper | n/a |
| reverse primer for Syk 1-99 5’-CTTGAGGAGGCAGACCTAGCCATCAGACTCCTG | This paper | n/a |
| forward primer for Syk 165-258 5’-GTCGACTCATGCCTTGGTTCCATGGAAAAATCTCT-3’ | This paper | n/a |
| reverse primer for Syk 165-258 5’- GCGGCCGCTGGGACAGTAAGAACTCTTAACAAACC-3’ | This paper | n/a |
| **Recombinant DNA** | | |
| pFastBac hVCAM-1-mIgG2a | Encysive Pharmaceuticals Inc. Houston, TX  Woodside, D.G. et al. 2006 | n/a |
| pGEX4T-Syk 1-116 | This paper | n/a |
| pGEX4T 1-163 | This paper | n/a |
| pGEX4T-Syk 1-99 | This paper | n/a |
| pGEX2T-Syk 6-370 | Shiue L., et al. (2) | n/a |
| pGEX4T-Syk 165-258 | This paper | n/a |
| **Software and Algorithms** | | |
| GraphPad Prism | GraphPad Software | https://www.graphpad.com/scientific-software/prism/ |
| FlowJo | FlowJo LLC | https://www.flowjo.com/ |
| Image J | NIH | <https://imagej.nih.gov/ij/>  RRID:SCR_003070 |
| Autodock vina | <http://vina.scripps.edu/> | RRID:SCR_011958 |
| Visual Molecular Dynamics | <http://www.ks.uiuc.edu/Research/vmd/> | RRID:SCR_001820 |
| PyMOL | <http://www.pymol.org/> | RRID:SCR_000305 |
| MATLAB | <http://www.mathworks.com/products/matlab/> | RRID:SCR_001622 |
| GROMACS | <http://www.gromacs.org/> | RRID:SCR_014565 |
| GRACE | <http://plasma-gate.weizmann.ac.il/Grace/> | 5.1.25-foss-2016a |
| **Other** | | |
| Complete protease inhibitor tablets | Roche | cat# 11873580001 |
| Glutathione Sepharose 4B beads | GE Life Sciences | cat# 17075601 |
| Quickchange II XL Site-Directed Mutagenesis Kit | Stratagene | cat# 200521 |
| pCR2.1 cloning vector | Novagen | cat# 69450-3 |
| pGEX2T | GE Healthcare | cat# 28954653 |
| pGEX4T | GE Healthcare | cat# 28954549 |
| Quick Start Bradford Protein Assay Kit | BioRad | cat# 5000202 |
| High Capacity NeutrAvidin agarose | ThermoFisher | cat# 29204 |
| 4-20% precast gels | BioRad | cat# 4561093 |
| 4-20% precast gels | Thermo Scientific | cat# 25204 |
| Protein G Sepharose 4 Fast Flow | GE Healthcare | cat# 17061801 |

**REFERENCES**

1. Woodside, DG, Kram, RM, Mitchell, JS, Belsom, T, Billard, MJ, McIntyre, BW, et al. Contrasting roles for domain 4 of VCAM-1 in the regulation of cell adhesion and soluble VCAM-1 binding to integrin alpha4beta1, *J Immunol*. (2006) 176:5041-9.

2. Shiue, L, Green, J, Green, OM, Karas, JL, Morgenstern, JP, Ram, MK, et al. Interaction of p72syk with the gamma and beta subunits of the high-affinity receptor for immunoglobulin E, Fc epsilon RI, *Mol Cell Biol*. (1995) 15:272-81.
